# Incubation of Lymphocytes with IL-2 Causes the Appearance of Antitumor T Cells Carrying FC, CD25, and LFA-1 on the Surface

**DOI:** 10.1134/S160767292270003X

**Published:** 2023-01-18

**Authors:** O. K. Ivanova, T. N. Sharapova, E. A. Romanova, D. V. Yashin, L. P. Sashchenko

**Affiliations:** Institute of Gene Biology of the Russian Academy of Sciences, Moscow, Russia

**Keywords:** Tag7, integrins, FasL, IL-2, tumor cells

## Abstract

To carry out antitumor activity against cells that have lost surface antigens, human lymphocytes must have a certain repertoire of surface proteins capable of contacting a tumor cell and inducing programmed cell death in it. In this work, we showed that activation of healthy donor cells by IL-2 cytokine within 6 days causes the appearance of FasL, CD25, and LFA-1 proteins on CD8+CD25+ T lymphocytes, and also converts the LFA-1 protein into an active form having a high affinity for its target, ICAM-1 integrin. The appearance of these proteins on the surface of this subpopulation of lymphocytes allows them to induce programmed cell death in HLA-negative tumor cells.

Cytotoxic lymphocytes are known to play a key role in antitumor protection. It is important to search for regulatory proteins that induce cytotoxic activity of lymphocytes, investigatethe the activation stages of these lymphocytes and mechanisms of tumor cell death under the action of these proteins. [1, 2] Understanding these processes will allow us to outline approaches of the direct regulation of tumor cell death.

We have recently shown that the ligand of the innate immunity receptor TREM-1, the Tag7 protein, not only enhances inflammatory processes, but also activates the generation of CD8+ T lymphocytes that kill MHC-negative tumor cells [3]. According to their phenotype, spectrum of target cells and mechanisms of induced cell death, the lymphocytes obtained by incubation with TREM-1 ligands were found to be identical to LAK cells [4]. The study of the activation stages of cytotoxic lymphocytes under the action of Tag7 allowed us to establish that IL-2 secretion is a key moment in the generation of these lymphocytes [3].

For a detailed analysis of the molecular mechanisms of TREM-1 dependent activation of cytotoxic lymphocytes, it is essential to detect changes in the phenotype of CD8+ T lymphocytes under the action of IL-2, which are associated with their cytotoxic effect.

We have previously shown that FasL, present on the surface of TREM-1–induced lymphocytes, is necessary for the induction of a cytotoxic signal in tumor cells [5]. We also found that the NKG2D receptor on the surface of the CD8+ T lymphocyte interacts with the non-canonical MHC molecule, the MicA protein present on the outer membrane of the tumor cell, and ensures that the lymphocyte recognizes thetarget cell. [6] The interaction of NKG2D with MicA is highly specific, but the stability of the intercellular complex NKG2D-MicA is quite low. To stabilize such a complex, it is necessary to form an immunosynapse, the creation of which involves adhesion molecules [7–9]. Adhesion molecules provide intercellular connections and their role in the formation of cellular complexes is extremely high. The proteins of the integrin family LFAI and ICAMI are the most well studied [10]. There are also references that glycoproteinsof the immunoglobulin family may play an important role for the adhesion of lymphocytic cells [11]. Integrins, taking part in the creation of immunosynapse are as necessary for the manifestation of cytotoxic action by lymphocytes, as proteins directly inducing cytotoxic processes in target cells.

The aim of this work was to study the effect of IL-2 on the composition of surface proteins of the lymphocyte fraction that performs antitumor cytotoxic activity.

Lymphocytes were obtained from the white cell-rich suspension of healthy donors by centrifugation on a Ficoll gradient according to a standard technique. All donors signed a voluntary consent, the material was accepted anonymously. CD8+, CD8+CD25+, and CD8+CD25- subpopulations of lymphocytes were isolated using kits with Dynabeads magnetic beads (Invitrogen, USA). To induce activation of cytotoxic lymphocytes, the total leukocyte fraction was incubated under standard conditions with the addition of IL2 (ThermoFisher Scientific, USA) at a concentration of 1000 u/ml for 6 days. K562 cells (human erythroblastoma) were cultured in RPMI medium (PanEco, RF) with the addition of 10% bovine embryonic serum (HyClone, USA). The culture was obtained from the bank of cell lines of the N.N. Blokhin Russian Research Center of the Russian Academy of Medical Sciences. To measure cytotoxicity, lymphocytes were added to K562 culture in a ratio of 20 : 1 and incubated for 20 hours. Cytotoxicity was measured using a Cytotox kit (Promega, USA) according to the manufacturer’s method.

The following antibodies were used for flow cytometry: antibodies to CD3 (Tri-Color), CD8 (APC), CD25 (PE/Cy7), CD56 (PerCP/Cy5.5), manufactured by Biolegend, USA, antibodies to FasL, NKG2D (FITC), ICAM1 manufactured by Santa Cruz Biotechnology, USA, antibodies to MICA, Fas manufactured by Sony, USA, secondary antibodies against Fc fragment of antibodies obtained in rabbit, labeled Alexa Fluor 546 and also obtained in mouse, labeled Alexa Fluor 488, manufactured by Life Technologies, USA. To study changes in the structure of LFA1, the antibody clone HI111 (labeled Alexa Flour 488) was used to detect the “closed” conformation, and m24 (manufactured by Biolegend, USA) was used for the “open”, active conformation. The cell phenotype was determined on a Cytoflex flow cytometer (Beckman Coulter Life Sciences, USA).

In cytotoxicity inhibition experiments, CD25 blocking antibodies at a concentration of 10 μg/mL (clone B-B10, BMS134, Invitrogen, USA), JAK inhibitors (JAK inhibitor I sc-204021, JAK3 inhibitor VI, ChemCruz, USA) were used.

For real-time PCR, the RNA of the samples was isolated in Trizol Reagent (Invitrogen, USA). cDNA synthesis was performed using Oligo(dT) primers (Eurogen, RF). The obtained products were used for qPCR with primers to the genes RPLP0, GAPDH, FasL, NKG2D, LFA1. RPLP0 and GAPDH mRNA levels were used as controls. Primers used: FasL forward: 5' GGATGTTTCAGCTCTTCCACC 3', reverse: 5' AGTTGGACTTGCCTGTTAAATGG 3'; NKG2D forward: 5' AATTCCCTTGACCGAAAGTTACTG 3', reverse: 5' AGTAAATCCTGGTCCTCTTTGCT 3'; GAPDH forward: CAACAGCGACACCCACTCCT, reverse CACCCTGTTGCTGTAGCCAAA; RPLP forward: 5′-ACTGGAGACAAAGTGGGAGCC, reverse: 5′-CAGACACTGGCAACATTGCG; LFA1: forward: 5' TGCTTACAATAAACTCTCCTCCAG 3', reverse: 5' TCGTAGGTGACTTTCAGGGT 3'. Measurements at each point were carried out in at least three replications and the average value was calculated. Expression levels were quantified using the 2ΔΔCt method.

The figures (unless otherwise indicated) present data from at least three independent experiments as an average ± standard deviation. The differences between processing and control were checked for significance using SigmaPlot (Systat Software Inc, Berkshire, UK) using a one-way ANOVA.

First, we investigated the effect of IL-2 on the activity of CD8+T lymphocyte subpopulations. PBMC of ten donors were analyzed and in all cases a significant increase in the CD8+CD25+ T lymphocyte subpopulation was detected (Fig. 1a). Using magnetic separation, subpopulations of CD8+CD25+- and CD8+CD25-T lymphocytes were isolated and their ability to induce cytolysis of MHC-negative tumor cells was determined. It can be seen that only the fraction of CD8+CD25+ – T lymphocytes has a cytotoxic activity (Fig. 1b).

**Fig. 1.**
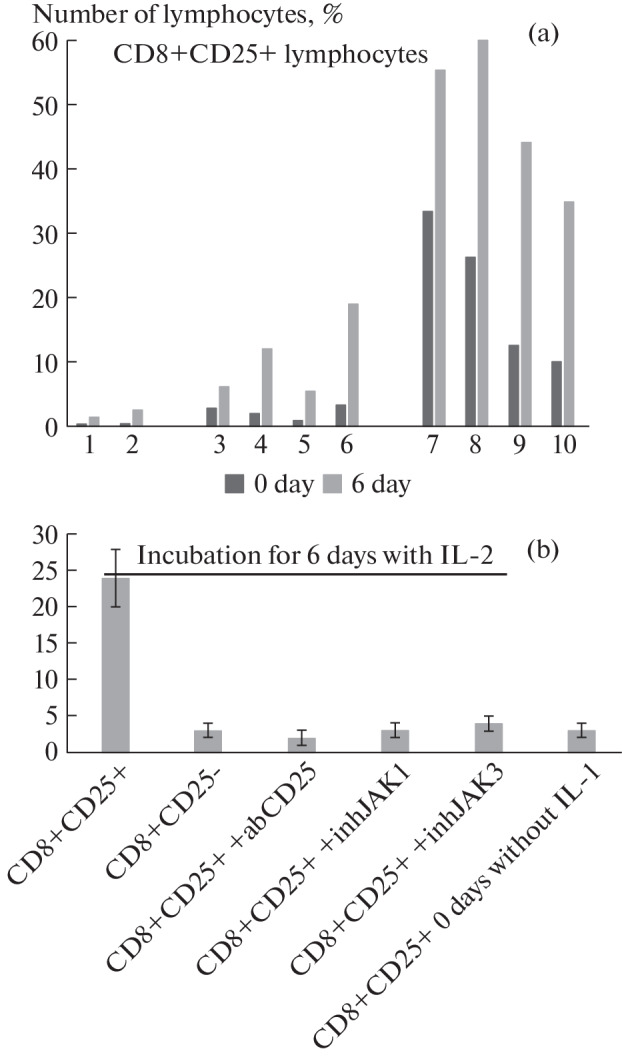
(a) Incubation of PBMC with IL2 causes an increase in the number of CD8+CD25+ T-lymphocytes. Experiments on material from 10 donors are presented. (b) Cytotoxic activity of CD8+ subpopulations of T cells in the presence of inhibitors and antibodies.

Earlier, we also found that the presence of the IL-2 receptor α-subunit (CD25 antigen) on the outer membrane of lymphocytes is essential for the cytotoxic activity of these lymphocytes. Incubation of PBMC with Tag7 protein in the presence of antibodies to CD25 antigen or removal from PBMC by magnetic separation and specific antibodies of CD25+-T lymphocytes led to the disappearance of cytotoxic activity [4]. We gave the following explanation to this effect: it was the result of blocking the interaction of IL-2 with its receptor and hindering the maturation of cytotoxic CD8+CD25+T lymphocytes under the action of this cytokine. Here we investigated the effect of anti-CD25 antibodies on the ability of already matured cytotoxic CD8+CD25+ T-lymphocytes to induce cytotoxic processes in tumor cells.

CD8+CD25+ T lymphocytes were incubated with antibodies and added to K562 cells. After 20 hours of coincubation, the number of dead tumor cells was calculated. It can be seen that anti-CD25 antibodies completely block cytotoxic activity (Fig. 1b). Interestingly, the addition of phosphokinase inhibitors JAK1 and JAK3—adapters that transmit a CD25-induced intracellular signal by activating the protein kinase cascade also blocked cytotoxic activity. Thus, the α-subunit of the IL-2 receptor participates in carrying out the intracellular signal necessary for the activation of cytotoxicity.

However, the presence of CD25-antigen on the surface of CD8+ T-lymphocyte is a necessary condition, but it is insufficient for the induction of cytotoxic processes. Comparison of cytotoxic activity of CD8+CD25+ T-lymphocytes isolated from PBMC of healthy donors without prior incubation with IL-2 and CD8+CD25+ T-lymphocytes obtained after 6-day incubation with IL-2 indicates that, despite the presence of CD25 protein on the surface of both subpopulations, incubation with IL-2 is necessary for cytotoxicity. Only CD8+CD25+ T lymphocytes incubated with IL-2 have cytotoxic activity (Fig. 1b).

In order to identify other proteins that cause the appearance of cytotoxicity, changes in the repertoire of proteins on the surface of CD8+ and CD8+CD25+ T-lymphocytes were investigated. Among these proteins were: NKG2D, responsible for binding to MicA on the surface of the tumor cell and recognition of the target cell, FasL, responsible for the induction of cytotoxic signal via FasL-Fas interaction. Recently, adhesion molecules that provide intercellular connections have attracted attention. It is known that during the interaction of cytotoxic lymphocytes with target cells, adhesion molecules are involved in the creation of immunosynapse [7]. From a fairly wide range of adhesion molecules, we investigated the role of integrin LFA1 and its ligand ICAM1, and glycoprotein from the family of immunoglobulins CD56.

Using the real-time PCR method, changes in the gene expression of antigens of interest were studied in CD8+ T lymphocytes incubated for 6 days with IL-2. It can be seen that the mRNA expression of CD25, FasL and LFA1 proteins increases in response to IL2 in a subpopulation of CD8+CD25+ cells (Table 1). Further, these proteins were studied on the surface of CD8+ and CD8+CD25+ T-lymphocytes using specific antibodies and flow cytometry (Figs. 2a, 2b). It is well known that during the interaction of the lymphocyte with the target cell, LFA1 changes the conformation, opening a highly affine binding site with ICAM1. The change in conformation ensures the formation of a strong immunosynapse necessary for cytotoxic effects [12].

**Table 1.** mRNA expression of CD25, FasL, NKG2D, LFA 1 proteins increases as a result of incubation of CD8+ il 2 T lymphocytes

	CD25*	FasL*	NKG2D	LFA1*
0 day	1	1	1	1
6 day	13.22 ± 2.53	35.88 ± 2.87	1.292 ± 0.12	1.42 ± 0.07

*p < 0.005.

**Fig. 2.**
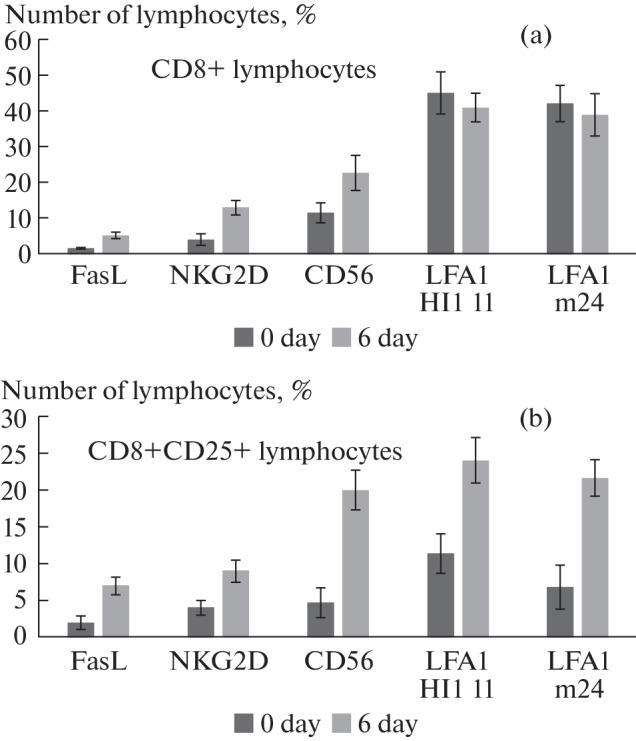
IL 2 causes a change in the phenotype of the CD8+ (a) and CD8+CD25+ (b) T-lymphocyte subpopulations.

In this work, we used antibodies detecting inactive (HI111) and active (m24) forms of LFA1.

It can be seen that FasL, NKG2D and CD56 are present on the surface of both CD8+ and CD8+CD25+ T-lymphocytes. After 6-day incubation with IL-2, the number of cells carrying these proteins on the surface increases. Integrin LFA1 is present on CD8+ and CD8+CD25+ T-lymphocyte membranes in both active and inactive forms. When we incubated CD8+ subpopulation of lymphocytes with IL2, we didn’t see significant changes in the fraction of LFA1+ cells, neither active, nor closed. However, the incubation of CD8+CD25+ subpopulation of lymphocytes with IL2 lead to rise of LFA1+ cells both in active and inactive forms (Figs. 2a, 2b).

It can be assumed that the formation of immunosynapse, as well as the presence of CD25, Fas and NKG2D, is absolutely necessary for the implementation of cytotoxic activity (Table 1).

To confirm this assumption, the effect of antibodies to the aforementioned proteins on the death of tumor cells under the action of these lymphocytes was studied (Fig. 3). Earlier we showed [6], and here we confirmed that antibodies to NKG2D and its MicA ligand, as well as antibodies to FasL and its Fas receptor completely block the cytotoxic effect of lymphocytes. It can also be seen that cytotoxic activity is significantly reduced in the presence of antibodies to the active form of LFA1. Cytotoxicity is completely blocked when lymphocytes are added to tumor cells previously incubated with antibodies to ICAM1. Antibodies to LFA1 in the “closed,” inactive conformation and to CD56 had no effect on the cytotoxicity of lymphocytes.

**Fig. 3.**
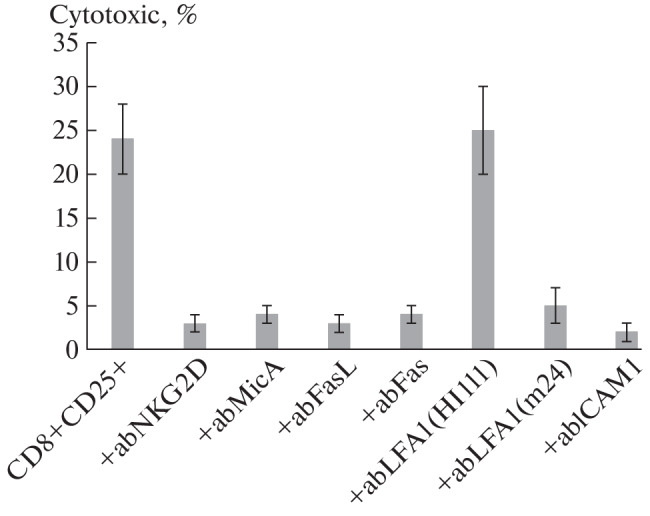
Cytotoxic activity of CD8+CD25+ T-lymphocytes in the presence of blocking antibodies.

Summarizing the above, it can be noted that the pivotal feature ofthe cytotoxically active population of CD8+CD25+ cells arising in PBMC after 6 days of incubation with cytokine IL-2 is a high percentage of cells carrying the proteins necessary for cytotoxic action: NKG2D, FasL, CD56 as well as the active form of LFA1. This helps the cells of this subpopulation to recognize the tumor cell, form a strong contact with it, and induce programmed cell death in it.
